# Intra-Arterial, but Not Intrathecal, Baclofen and Codeine Attenuates Cough in the Cat

**DOI:** 10.3389/fphys.2021.640682

**Published:** 2021-03-05

**Authors:** Wendy L. Olsen, Melanie Rose, Frank J. Golder, Cheng Wang, Julie C. Hammond, Donald C. Bolser

**Affiliations:** Department of Physiological Sciences, University of Florida, Gainesville, FL, United States

**Keywords:** baclofen, codeine, airway protection, cough, intrathecal, antitussives, opioid, GABA-B receptor agonist

## Abstract

Centrally-acting antitussive drugs are thought to act solely in the brainstem. However, the role of the spinal cord in the mechanism of action of these drugs is unknown. The purpose of this study was to determine if antitussive drugs act in the spinal cord to reduce the magnitude of tracheobronchial (TB) cough-related expiratory activity. Experiments were conducted in anesthetized, spontaneously breathing cats (*n* = 22). Electromyograms (EMG) were recorded from the parasternal (PS) and transversus abdominis (TA) or rectus abdominis muscles. Mechanical stimulation of the trachea or larynx was used to elicit TB cough. Baclofen (10 and 100 μg/kg, GABA-B receptor agonist) or codeine (30 μg/kg, opioid receptor agonist) was administered into the intrathecal (i.t.) space and also into brainstem circulation *via* the vertebral artery. Cumulative doses of i.t. baclofen or codeine had no effect on PS, abdominal muscle EMGs or cough number during the TB cough. Subsequent intra-arterial (i.a.) administration of baclofen or codeine significantly reduced magnitude of abdominal and PS muscles during TB cough. Furthermore, TB cough number was significantly suppressed by i.a. baclofen. The influence of these drugs on other behaviors that activate abdominal motor pathways was also assessed. The abdominal EMG response to noxious pinch of the tail was suppressed by i.t. baclofen, suggesting that the doses of baclofen that were employed were sufficient to affect spinal pathways. However, the abdominal EMG response to expiratory threshold loading was unaffected by i.t. administration of either baclofen or codeine. These results indicate that neither baclofen nor codeine suppress cough *via* a spinal action and support the concept that the antitussive effect of these drugs is restricted to the brainstem.

## Introduction

Cough is a protective behavior meant to remove irritant materials from the airways with explosive bursts of air and is characterized by a large and coordinated increase in activity in inspiratory and expiratory muscles (Ross et al., 1955; Widdicombe, 1980). However, pathological factors can modify respiratory function and increase the frequency and persistence of cough, which can lead to significant morbidity. As a result, antitussives are used to ameliorate symptoms of chronic cough (Aylward et al., 1984). However, in a large number of human studies antitussives often lead to unpleasant side effects and there is a large demand for additional research on safer and more effective cough suppressants (Dicpinigaitis et al., 1997, 2014; Dicpinigaitis, 2009; Smith et al., 2012).

Antitussives are thought to inhibit cough by acting in the brainstem at a central site of action (Keller et al., 2017; Korpás and Tomori, 1979; Mutolo et al., 1985; Satia et al., 2016). These drugs work through a central mechanism by inhibiting a functionally identified segment of the brainstem control mechanism for cough termed a gate. The gate is proposed to control the expression of tracheobronchial (TB) cough. Given that laryngeal cough is relatively insensitive to suppression by intravenous (i.v.) codeine (Keller et al., 2017; Korpás and Tomori, 1979; Mutolo et al., 1985; Satia et al., 2016), the gating hypothesis does not extend to coughing induced from the larynx. However, cough from this organ may have a similar control mechanism, but if so, it is insensitive to antitussives. The gating mechanism is proposed to consist of specialized populations of neurons that directly excite the respiratory/cough pattern generator and modulate the expiratory motor activity related to cough (Bolser and Davenport, 2002, 2007; Bolser et al., 2006; Bolser, 2006). Specifically, there are interneurons located in the nucleus tractus solitarious (NTS) that affect afferent information, premotor inspiratory and expiratory neurons in the medulla, and expiratory and inspiratory motoneurons in the spinal cord (Bolser and Davenport, 2002). Intrathecal (i.t.) administered antitussives may, in part, have an effect on cough through the expiratory and inspiratory motoneurons *via* the spinal cord. This alternative route of administration could alleviate negative reported side effects.

While a brainstem site of action for antitussive drugs is generally accepted, there is no information regarding potential direct actions of these drugs on spinal circuits controlling chest wall and abdominal motor drive to respiratory muscles. Suppression of cough-related motor drive, especially to abdominal muscles, is a prominent feature of the effects of these drugs in animal models (Bolser, 1985; Poliacek et al., 1985, 2010; Bolser et al., 1993, 1997, 1999; Mutolo et al., 2008; Castillo and Pitts, 2013; Xu et al., 1985). We hypothesized that antitussive drugs would act, in part, to suppress abdominal motor drive during cough through an action in the spinal cord. We tested this hypothesis by administering two well-known cough suppressants, codeine and baclofen, *via* the i.t. route in a feline model of cough. Codeine was chosen because it is a standard cough suppressant drug in animal models and is one of the most widely prescribed antitussives in humans (Schappert, 1999). The GABA-B receptor agonist, baclofen, was chosen because it has antitussive activity in animal models (Bolser et al., 1993; Bolser et al., 1994; Canning et al., 2012) and humans (Dicpinigaitis et al., 1998). Further, baclofen was originally introduced in the 1960’s to treat spasticity *via* an action through a GABA system potentially in the spinal cord (Bowery, 1982). While the central actions of baclofen in the brainstem was thoroughly studied in animal models (Dicpinigaitis et al., 1997; Bolser et al., 1999; Bolser and Davenport, 2002, 2007; Bolser, 2006; Canning et al., 2012; Korpás and Tomori, 1979), to our knowledge a potential spinal action of this drug to suppress cough has never been studied.

## Materials and Methods

The experiments, procedures, and method of euthanasia described herein was approved by the University of Florida Animal Care and Use Committee and were performed in accordance with the *Guide for the Care and Use of Laboratory Animals*. Twenty-two (2.0–5.0 kg) spontaneously breathing cats were anesthetized with sodium pentobarbital (35 mg/kg, i.v.). Sixteen of 22 were male and six were female. End-tidal CO_2_ (Datex Engstrom, *Capnomac Ultima*; ET_CO2_) was monitored, and supplemental doses of anesthetic (0.1 mg/kg, i.v.) were administered when the ET_CO2_ dropped below 3.9%. Additionally, palpebral reflexes, jaw tone, and blood pressure [Becton Dickenson (B-D) – Model P23XL] were continuously monitored to maintain appropriate anesthesia levels of the animals. In the case of animal responsiveness to reflexes and/or jaw tone, the animal was administered supplemental doses of anesthetic and the experiment was re-tested before proceeding with experimental procedures. Catheters were placed in a femoral artery and vein for monitoring blood pressure, arterial gases (Abott Labs, *I-stat*) and administering drugs, respectively. Atropine sulfate (0.1 mg/kg i.v.) was administered to block reflex tracheal secretions. This dose was informed by veterinarians and is consistent with doses of those used in veterinary clinical practice used to reduce reflex mucus secretion (Plumb, 2011). A three-way tracheal cannula was inserted through an incision at the fourth tracheal segment. Body temperature was maintained at 37 ± 1°C with a homoeothermic blanket system (Harvard Apparatus).

### Animal Preparation

An incision was made in the left forelimb. The brachial plexus was exposed and the costocervical, thyrocervical, and internal thoracic branches of the axillary artery were identified and clamped. A cannula was placed in the axillary artery and its tip moved to the vertebral arterial branch. Bipolar plastic-coated stainless steel wire electrodes were placed in the parasternal (PS), rectus abdominis, and transversus abdominis (TA) muscles according to the technique of Basmajian and Stecko (Basmajian and Stecko, 1962). Electrodes were placed in the PS muscle at T_3_. The rectus abdominis muscle electrodes were placed through a small incision in the skin, approximately 7 cm caudal to the xiphoid process and 1 cm lateral to the midline.

### Recording Procedures

Electrodes were placed in TA muscles through an incision in the left lower abdominal skin, midway between the midline and the axillary line. Electromyograms (EMGs) from these muscles were amplified (Grass, Model *P511*), band-pass filtered (0.1–5.0 kHz), monitored on an oscilloscope, and integrated with a resistance-capacitance circuit (100-ms time constant; Cambridge Electronic Design, 1,401 Mrk II) and recorded using Cambridge Electronic Design, Spike II software.

For i.t. injections, animals were placed prone in a stereotaxic frame with their head elevated above the spinal column and remained in the frame for the duration of the experiment. An incision was made just over the lumbosacral space. An 18-gage, 3-inch spinal needle was placed just caudal to L7 and advanced through the dura. The stylet was examined for flow of cerebrospinal fluid (CSF) to confirm subdural placement. A 19-gage cannula (B-Braun Medical Inc.,–Open-tip) was advanced approximately 5 cm through the stylet into the subdural space for the i.t. administration of vehicle, baclofen, or codeine. Placement of the cannula was verified postmortem.

Cough was defined as an inspiratory-related burst of EMG activity in the PS muscle immediately followed by a burst of EMG activity in the abdominal muscles. Cough was elicited by mechanical stimulation of the intrathoracic trachea with a small length of polyethylene tubing (PE90) into the intrathoracic trachea *via* the tracheal cannula. This catheter was inserted into the trachea for 10 s (rotated at approximately 2 Hz) to elicit repetitive coughing. Breathing was monitored by the EMG activity of the PS, rectus abdominis, and TA muscles and continuous ET_CO2_ monitoring.

### Protocol

A series of 10 s mechanical stimuli was applied to the intrathoracic airways were performed with a total of 1 min elapsed time between stimulus trials to establish a stable baseline for TB cough. The antitussive activity of baclofen was evaluated from cumulative dose-response relationships (10 and 100 μg/kg) after i.t. and successive intra-arterial (i.a.) (10 and 100 μg/kg) administration. The antitussive activity of codeine was also evaluated at doses of 30 μg/kg for i.t. and 30 μg/kg i.a. administration in a separate group of animals. Additionally, vehicle (artificial CSF for i.t. and physiological saline for i.a.) was administered. The highest doses chosen for each drug represented ED50-70 values established in this model in a previous study (Bolser et al., 1999).

#### Stimulation Protocol for Baclofen and Codeine

Following the i.t. doses, 5–7 min were allowed to elapse before trial commencement. Stimulus trials were applied at 1 min intervals for a total of five stimulus trials between i.t. doses. Approximately 60 s following the cough trial series, a non-rebreathing valve was attached to the tracheal cannula. An expiratory load of 15 cm H_2_O was applied by attaching a hose to the expiratory port of the valve and submerging the end of the hose in a reservoir of water. Each load was 1 min in duration. Approximately 60 s following the expiratory load, a noxious mechanical stimulus was applied to the base of the tail by clamping down with a hemostat for a duration of 60 s. The hemostat was rotated in the horizontal plane by approximately 45 degrees at a frequency of 0.5 Hz for the duration of the trial. After the completion of the noxious stimulus, approximately 1 min lapsed before beginning the next i.t. dose. Once the i.t. doses had been administered, the i.a. protocol commenced. Following the i.a. doses, 5–7 min were allowed to elapse before trial commencement. Stimulus trials for TB cough, expiratory load, and noxious mechanical stimulus were applied similarly as in the i.t. administration. Codeine trials occurred similarly. The noxious mechanical stimulus was not applied in the codeine protocol because we were aware of codeine’s effective dosages.

### Compounds

Compounds used in this study include atropine sulfate (Sigma Chemical Co., St. Louis, MO, United States), artificial Cerebral Spinal Fluid (CSF) (Harvard Apparatus, Holliston, MA, United States), codeine phosphate (Sigma Chemical Co., St. Louis, MO, United States), and baclofen (Research Biochemicals, Natick, MA, United States). Baclofen (i.a.), codeine (i.a.), and atropine were dissolved in physiological saline. Artificial CSF was employed as the vehicle for i.t. administration of baclofen and codeine.

### Statistics

The data were analyzed using SigmaPlot 13.0 (Systat Software, Inc.). The data are expressed as mean ± s.e. mean. A student’s *t*-test, one-way ANOVA, or two-way ANOVA was used to evaluate differences between means. If a normality test (Shapiro–Wilk, *p* < 0.05) was failed, an ANOVA on ranks was conducted. Differences between means were considered significant if *p* < 0.05, and were followed by *post hoc* pair wise comparisons (Holm-Sidak method if normality was not violated and Dunn’s test if an ANOVA on ranks test was conducted). The EMG amplitudes of the three largest bursts during each expiratory load were averaged to obtain a single value for each load. The EMG burst amplitudes during expiratory loading, tail pinch and coughing were expressed as a percentage of the average of the pre-vehicle amplitudes. EMG activity during the tail pinch stimulus was analyzed by calculating the area of abdominal EMG activity during the 60-second stimulus then comparing the differences in area. Cardiorespiratory results for both baclofen and codeine are discussed in Table 1.

**TABLE 1:** Influence of baclofen and codeine on cough and cardiorespiratory parameters.

Baclofen					

	Vehicle	10 μg/kg i.t.	100 μg/kg i.t.	10 μg/kg i.a.	100 μg/kg i.a.
CT_I_	0.74 + 0.07 s	0.67 + 0.14 s	0.67 + 0.14 s	0.65 + 0.18 s	0.75 + 0.10 s
CT_E_	0.91 + 0.06 s	0.84 + 0.17 s	0.80 + 0.17 s	0.81 + 0.16 s	0.97 + 0.11 s
CT_TOT_	1.64 + 0.38 s	1.51 + 0.31 s	1.47 + 0.31 s	1.47 + 0.33 s	1.72 + 0.21 s
BP	143.67 + 5.80	135.0 + 5.47	124.0 + 6.40	126.50 + 5.92	131.28 + 6.55
PS_AMP_	100.0	100 + 6	95 + 5	93 + 7	104 + 14
T_I_	1.61 + 0.26	1.19 + 0.20	1.32 + 0.26	1.39 + 0.14	1.30 + 0.26
T_E_	1.35 + 0.20	1.47 + 0.25	1.62 + 0.24	1.50 + 0.26	1.45 + 0.20
pH		7.38 + 0.02		7.39 + 0.01	
pCO_2_		28.9 + 0.50		29.6 + 0.90	
pO_2_		101.7 + 1.90		100.2 + 1.90	
ETCO_2_		31.7 + 1.40		29.4 + 0.70	
HR_Min_	207.33 + 8.03	210.67 + 9.62	211.67 + 9.65	218.33 + 8.85	222.33 + 8.86
RR_Min_	22.5 + 2.16	23.5 + 2.50	23.2 + 2.95	24.0 + 1.55	25.5 + 2.85

**Codeine**					

	**Vehicle**		**30 μg/kg i.t.**		**30 μg/kg i.a.**

CT_I_	0.70 + 0.10 s		0.61 + 0.14 s		0.83 + 0.27 s
CT_E_	1.04 + 0.18 s		0.85 + 0.17 s		1.27 + 0.56 s
CT_TOT_	1.74 + 0.55 s		1.45 + 0.36 s		2.09 + 0.84 s
CT_TOT_	1.74 + 0.55 s		1.45 + 0.36 s		2.09 + 0.84 s
BP	139.80 + 11.10		133.80 + 15.50		130.40 + 18.90
PS_AMP_	1.0		94 + 5		90 + 8
T_I_	1.49 + 0.44		1.28 + 0.32		1.45 + 0.79
T_E_	1.42 + 0.58		1.32 + 0.42		1.51 + 0.36
Ph			7.39 + 0.01		7.38 + 0.01
pCO_2_			32.0 + 1.49		30.4 + 2.30
pO_2_			103.4 + 2.84		104.2 + 8.55
ETCO_2_			31.0 + 1.48		32.0 + 1.67
HR_Min_	225.6 + 9.02		226.80 + 8.98		280.32 + 13.50
RR_Min_	24.0 + 3.29		25.8 + 2.62		17.8 + 2.69

*A one-way ANOVA was conducted for the cough phase analysis. Cough phases were not significantly different for baclofen or codeine at any dose. Blood pressure (BP), parasternal amplitudes (PS_*Amp*_), respiratory rate (RR), heart rate (HR), total inspiratory (T_*I*_) time, and total expiratory (T_*E*_) time were analyzed using a one-way ANOVA. No significant differences were observed at any dose. pH levels, pCO_2_, pO_2_, and ETCO_2_ for each administration route (intrathecal and intra-arterial) were subjected to a Student’s *t*-test. No significant differences were revealed. The number of animals for baclofen and codeine-treated animals were 6 and 5, respectively. Data are expressed as mean ± s.e. mean.*

## Results

There were 22 animals used for this study. A total of 4,015 TB coughs were evaluated in six animals who received baclofen, five animals who received codeine, and four control animals (15 total). All six animals that were administered baclofen also were challenged with expiratory threshold loading and noxious mechanical stimulation of the tail. Seven additional animals were evaluated with noxious stimulation of the tail but were not challenged with TB cough (one administered baclofen, six administered vehicle). For codeine administration, four of five animals that were challenged with TB cough were also challenged with expiratory threshold loading and none were challenged with noxious mechanical stimulation of the tail.

### Baclofen

**FIGURE 1:**
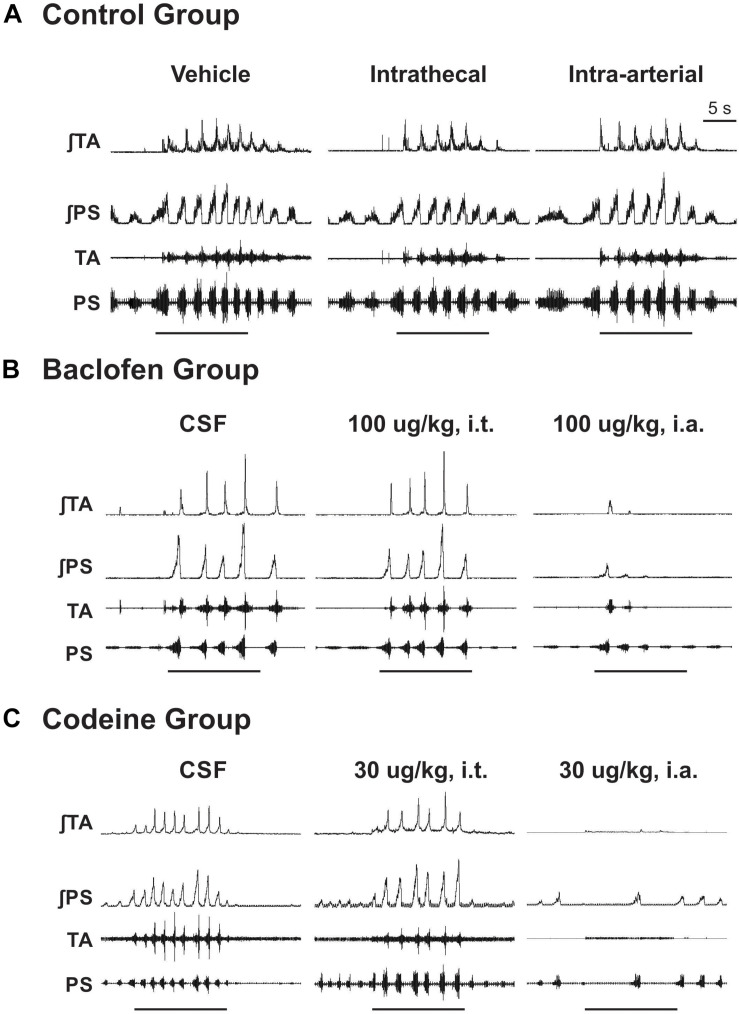
Examples of EMG responses of parasternal and transversus abdominis muscles during cough in a single animal. **(A)** CSF and saline adminstration, **(B)** baclofen administration, and **(C)** codeine administration. TA, transversus abdominis muscle EMG; PS, parasternal muscle EMG. Horizontal bars are periods of cough trials.

**FIGURE 2:**
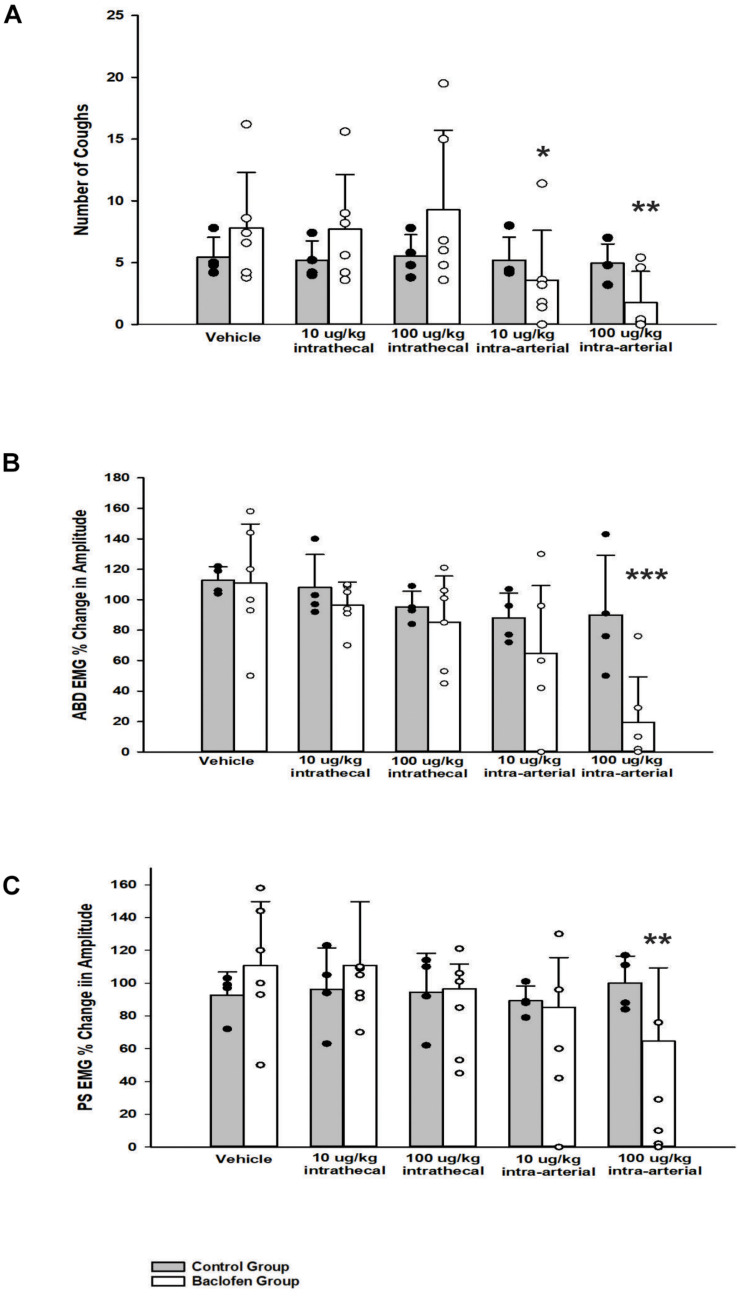
Influence of baclofen on **(A)** the number of tracheobronchial cough, **(B)** the magnitude of transversus abdominis muscle during tracheobronchial cough, and **(C)** the magnitude of parasternal muscle during tracheobronchial cough. A two-way ANOVA revealed a significant difference between the control and baclofen group **(A)** *p* = 0.008, **(B)** *p* = 0.013, and **(C)** *p* = 0.007. The baclofen group had fewer number of coughs and smaller magnitudes of the transversus abdominis and parasternal muscles during tracheobronchial cough when compared to the control group. Specifically, a one-way ANOVA revealed the baclofen group had a significant reduction in: number of coughs at the 10 μg/kg i.a. (*p* = 0.049) and the 100 μg/kg i.a. dose (*p* = 0.004), magnitude of the transversus abdominis muscle at the 100 μg/kg i.a. dose (*p* < 0.001), and magnitude of the parasternal muscle at the 100 μg/kg i.a. dose (*p* = 0.001). Each bar represents the mean ± s.e.m of six animals who received baclofen (Baclofen Group) and 4 control animals (Control Group). Cough numbers were normalized by totaling the number of coughs and dividing by the number of trials. Amplitudes were normalized by magnitudes observed in the vehicle period. An asterisk (*p < 0.05, **p < 0.005, ***p < 0.001) indicates a significant reduction in amplitude in cough and amplitude relative to vehicle and the Control Group.

Abdominal and PS muscle EMGs were strongly activated during repetitive coughing (Figure 1). A one-way ANOVA revealed that i.t. baclofen had no significant effect on the number of TB coughs (Figure 2), at 10 μg/kg (*p* = 0.97) or 100 μg/kg (*p* = 0.82), relative to vehicle for the baclofen group. Further, i.t. baclofen did not significantly affect abdominal EMG amplitude during TB cough (Figure 2) at either dose (10 μg/kg, *p* = 0.71 or 100 μg/kg, *p* = 0.53) or PS EMG amplitude at either dose (Figure 2; 10 μg/kg, *p* = 0.75 or 100 μg/kg, *p* = 0.86). There were no significant effects of i.a. baclofen at the 10 μg/kg dose (Figure 2; *p* = 0.16 on abdominal or PS (Figure 2; *p* = 0.76) EMG activity relative to vehicle. A two-way ANOVA was conducted to determine if differences existed between the animals who received baclofen and the control group (animals who received CSF i.t. and saline i.a.) at different doses for number of coughs, abdominal, and PS EMG magnitudes. Results revealed a Group x Dose interaction for the PS EMG condition (*p* = 0.007). A *post hoc* comparison determined that the baclofen group had reduced magnitudes of the PS muscles at the 100 μg/kg i.a. dose when compared to the control group (Figure 2). Additionally, results indicated that the baclofen group did have significantly reduced magnitudes of the TA (*p* = 0.013) and fewer number of coughs (*p* = 0.008) during TB cough trials, compared to the control group. i.a. administration of baclofen did significantly decrease abdominal (*p* < 0.001) and PS (*p* < 0.001) EMG activity at the 100 μg/kg dose when compared to vehicle. Further, i.a. administration of baclofen decreased TB cough number at the 10 μg/kg dose (*p* = 0.049) and the 100 μg/kg dose (*p* = 0.004), relative to vehicle.

**FIGURE 3:**
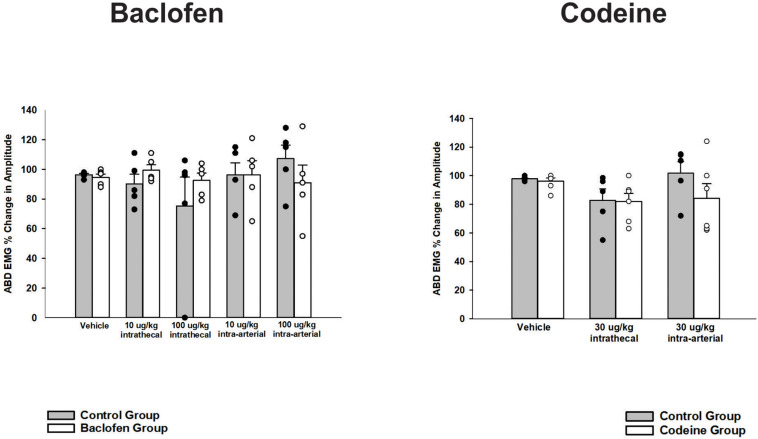
Influence of baclofen and codeine on responses of the transversus abdominis muscle EMG to expiratory threshold loads. Each graph contains a control condition where the gray bar represents the mean change in amplitude and the black circles are plotted individual data. The white bar is indicative of the mean responses during the drug intervention (i.e., baclofen or codeine as indicated by the title) and each white circle is the plotted individual data. Neither intrathecal or intra-arterial baclofen or codeine altered transversus abdominis (TA) magnitudes during expiratory threshold loading. Values are normalized maximum TA magnitudes during expiratory threshold loads of 15 cm H2O. Each bar represents the mean ± s.e. mean of five animals for baclofen (*p* = 0.43), five animals for codeine (*p* = 0.31), and four control-treated animals using a two-way ANOVA.

A one-way ANOVA was conducted for a cough phase analysis at vehicle and each dose of baclofen. Values for inspiratory, expiratory and total cough phase duration means and their s.e.m.’s are reported in Table 1 (Wang et al., 2009). Results indicated that there were no significant differences for i.t. or i.a. administration at any dose (*p* = 0.095). During expiratory threshold loading, there was no significant difference in the magnitude of abdominal EMG burst activity during the i.t. or i.a. administration of baclofen at any dose (*p* = 0.93; Figure 3).

**FIGURE 4:**
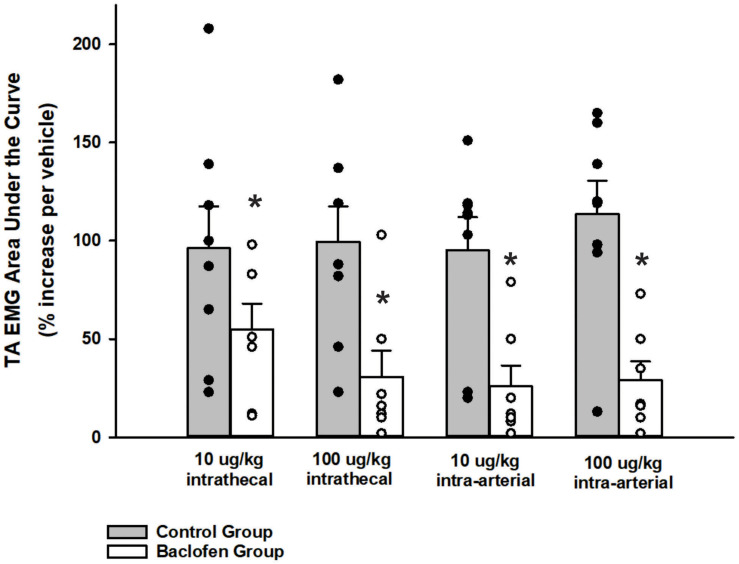
Influence of baclofen on the magnitude of transversus abdominis EMGs during noxious pinch of the tail. A two-way ANOVA revealed a significant difference between the baclofen and control group, *p* < 0.001. Intrathecal baclofen significantly reduced the transversus abdominis (TA) EMG during noxious pinch of the tail at both doses. Subsequent intra-arterial administration of baclofen did not reduce this EMG further. Each bar represents the mean ± s.e. mean of seven animals who received baclofen (10 μg/kg i.t., *p* < 0.001; 100 μg/kg i.t., *p* < 0.001; 10 μg/kg i.a., *p* < 0.001; 100 μg/kg i.a., *p* < 0.001) and 8 control animals using a one-way repeated measures ANOVA Amplitudes were normalized to those observed in the vehicle trials. Stimulus was applied for approximately 40 s. An asterisk (*p < 0.001) indicates a significant reduction in amplitude relative to vehicle control.

A two-way ANOVA was conducted to determine if differences existed between the animals who received baclofen and the control group (animals who received CSF i.t. and saline i.a.) at different doses for noxious somatic stimulation (tail pinch stimulus). Results indicated that differences existed between the control and baclofen group, *p* < 0.001. For animals who received baclofen, a one-way ANOVA revealed i.t. and i.a. administration of baclofen did significantly reduce abdominal motor activity induced by a noxious somatic stimulus applied to the base of the tail at the 10 μg/kg (*n* = 7; i.t. *p* = 0.005; i.a. *p* < 0.001; Figure 4) and 100 μg/kg doses (*n* = 7, i.t. and i.a. *p* < 0.001; Figure 4) when compared to vehicle administration. The suppressive effect of baclofen on abdominal EMG responses to noxious somatic stimulation indicates that the i.t. and i.a. doses of baclofen chosen were effective when compared to a vehicle intervention (*n* = 7; *p* < 0.001, i.t. and *p* < 0.001 i.a; Figure 4).

### Codeine

**FIGURE 5:**
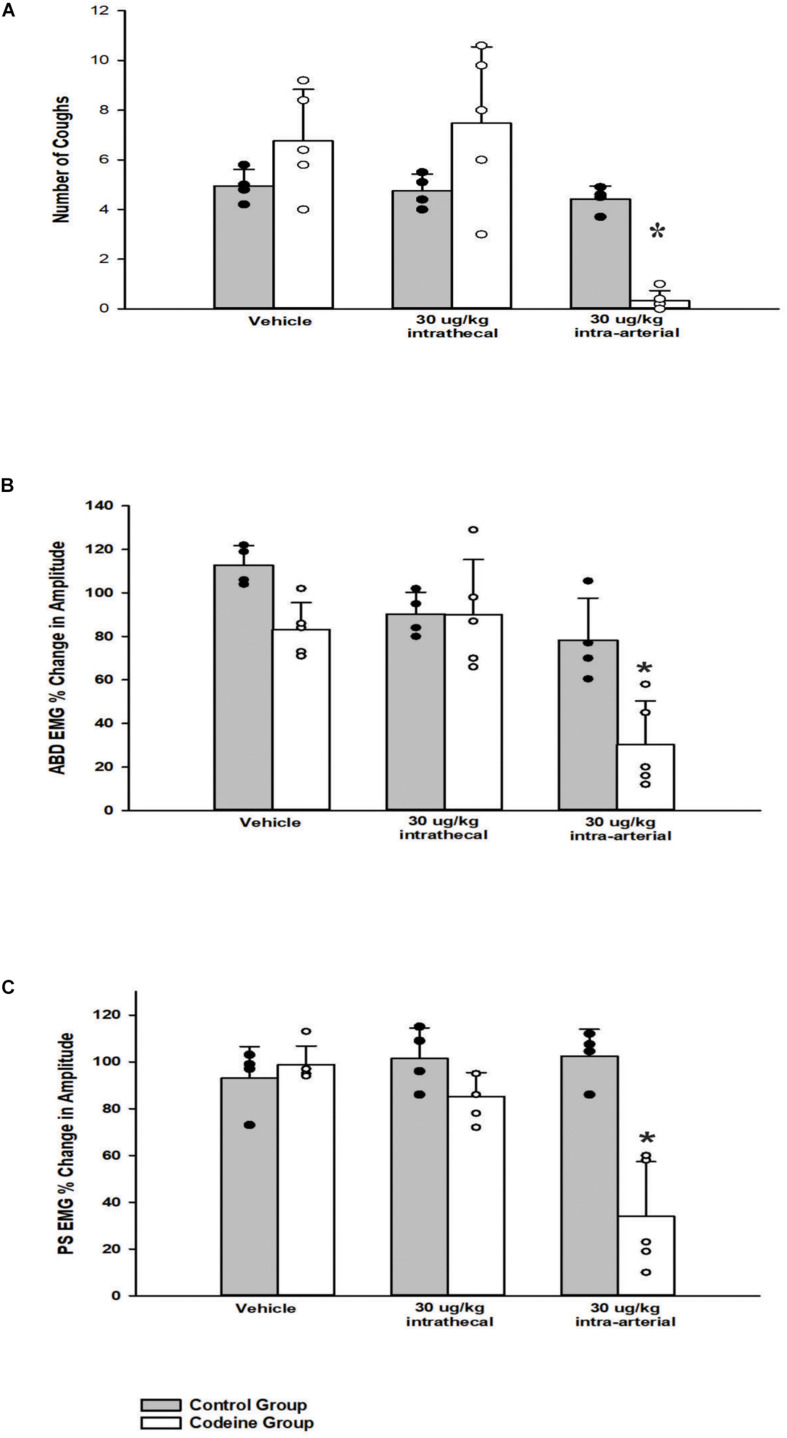
Influence of codeine on **(A)** the number of tracheobronchial cough, **(B)** the magnitude of transversus abdominis muscle during tracheobronchial cough, and **(C)** the magnitude of parasternal muscle during tracheobronchial cough. A two-way ANOVA revealed a Group x Dose interaction for **(A)** *p* < 0.001, **(B)** *p* = 0.030, and **(C)** *p* < 0.001. The codeine group had fewer number of coughs and smaller magnitudes of the transversus abdominis and parasternal muscles during tracheobronchial cough at the 30 μg/kg i.a. dose, **(A)** *p* < 0.001, **(B)** *p* < 0.001, and **(C)** *p* < 0.001. Each bar represents the mean ± s.e.m. of five animals who received codeine (Codeine Group) and four control animals (Control Group). Cough numbers were normalized by totaling the number of coughs and dividing by the number of trials. Amplitudes were normalized by magnitudes observed in the vehicle period. An asterisk (*p < 0.001) indicates a significant reduction in amplitude in cough and amplitude relative to vehicle and the Control Group.

A one-way ANOVA revealed that i.t. administration had no significant effect (30 μg/kg, *n* = 5; *p* = 0.61) on TB cough, relative to vehicle. i.t. administration of codeine did not significantly affect abdominal (*n* = 5; *p* = 0.60) or PS (*n* = 5; *p* = 0.19) EMG magnitudes at 30 μg/kg. Conversely, a two-way ANOVA was conducted to determine if differences existed between the animals who received codeine and the control group (animals who received both CSF i.t. and saline i.a.) at different doses for number of coughs, abdominal, and PS EMG magnitudes. Results revealed a Group x Dose interaction for number of coughs (*p* < 0.001), magnitudes of the TA (*p* = 0.030), and PS (*p* < 0.001) muscles. A *post hoc* pairwise comparison indicated that the codeine group did have significantly reduced number of coughs (*p* < 0.001), magnitudes of the TA (*p* < 0.001), and PS (*p* < 0.001) muscles during TB cough trials at the 30 μg/kg i.a. dose, compared to vehicle and the control group (Figure 5).

A one-way ANOVA was conducted for a cough phase analysis at vehicle and each dose of codeine. Values for inspiratory, expiratory and total cough phase duration means and their s.e.m.’s are reported in Table 1 (Wang et al., 2009). Results indicated there were no significant differences for codeine on cough phase durations when administered i.t. or i.a. (*n* = 5; *p* = 0.303). During expiratory threshold loading, there was no significant difference in abdominal EMG burst activity during the i.t. or i.a. administration of codeine at any dose (*n* = 4; *p* = 0.31; Figure 3).

## Discussion

The main findings of this study are that i.t. administration of baclofen and codeine did not significantly inhibit TB cough but i.a. delivery of these drugs reduced both cough number and the magnitude of expiratory muscle EMGs during coughing. Neither baclofen nor codeine suppressed abdominal motor drive during expiratory threshold loads. However, the abdominal EMG response to a noxious stimulus applied to the base of the tail was significantly inhibited by i.t. administration of baclofen.

Baclofen has been shown to suppress coughing in animals and humans (Bolser et al., 1994, 1999; Canning et al., 2012; Dicpinigaitis et al., 1997) and codeine also suppresses cough in animal models (Bolser et al., 1995; Chou and Wang, 1975; May and Widdicombe, 1954), but the antitussive activity of codeine in humans has been questioned recently (Bolser and Davenport, 2007; Smith et al., 2006). Both drugs have central antitussive properties that reduce the number and magnitude of coughing in animal studies (Bolser et al., 1993, 1994, 1995; Bolser and DeGennaro, 1994; Simera et al., 2010). We and others demonstrated that effective dose ratios generated from i.v. and i.a. (vertebral artery) dose-response relationships can differentiate between the cough suppressant actions of drugs that have solely a peripheral site of action from those with a central component (Bolser et al., 1995; Chou and Wang, 1975). Doses of centrally active antitussive drugs when administered *via* the brainstem circulation are comparatively low relative to i.v administration because of direct delivery to their site of action (Bolser et al., 1995). Our prior work showed that baclofen and codeine have high effective dose ratios (Bolser et al., 1993, 1994, 1995) in the cat and we suggested that the brainstem is a major site of action to suppress cough for these drugs. The evidence for central antitussive actions of these drugs also includes studies that utilized direct administration to the brainstem by microinjection or intracerebroventricular administration (Mutolo et al., 1985, 2008; Ohi et al., 2005; Poliacek et al., 2010, 2012; Mutolo, 2017; Poliacek et al., 2017). The results of these studies convincingly attributed the actions of these drugs to selected areas of the brainstem. On balance, the evidence supports actions of these drugs at multiple sites in the brainstem. As such, administration of baclofen, codeine or other centrally-action cough suppressant drugs by vascular routes would be expected to affect the excitability of cough-related neurons in several areas of the brainstem. However, these drugs also enter the spinal cord after systemic administration. Potential actions of these cough suppressant drugs in the spinal cord would be expected to be limited to suppression of motor drive to respiratory muscles resulting in weaker coughs. The number of coughs and their temporal features are regulated by brainstem neuronal circuits (Shannon et al., 1996, 1998, 2000, 2004a; Bongianni et al., 1998; Baekey et al., 2003; Shannon et al., 2004b). Our present results do not support a spinal action on cough-related motor pathways by codeine or baclofen. While peripheral actions of these drugs on cough following systemic administration are possible (Gruhzit, 1957), it is likely that the major actions of these drugs on motor features of coughing are restricted to the brainstem, at least in animal models. We cannot rule out actions of these drugs on suprapontine pathways that participate in coughing (McGovern et al., 2015a; McGovern et al., 2015b). Indeed, suprapontine areas present strong contributions to the genesis of urge-to-cough in humans (Mazzone et al., 2007, 2009, 2011, 2013; Mazzone et al., 2015).

When administered to the brainstem circulation, baclofen and codeine significantly inhibited TB cough. These findings are consistent with the findings of Bolser et al. (1993), Bolser and DeGennaro (1994), Bolser et al. (1994), and Canning et al. (2012). For both baclofen and codeine, cough number decreased by more than 60% when administered intra-arterially, relative to vehicle and control. Cough-related abdominal muscle EMG activity was depressed by i.a. administration of either baclofen or codeine. PS inspiratory muscle activity was significantly depressed during cough at the highest dose of baclofen (100 μg/kg i.a.) and codeine administration (30 μg/kg i.a.). These finding represent a significant departure from the results of previous studies in our laboratory that diaphragm muscle EMG activity during TB coughing was not affected by i.a. administration of codeine at this dose (Castillo and Pitts, 2013; Xu et al., 1985). For baclofen, previous work has shown that doses up to 30 mg/kg, i.a. had no effect on diaphragm motor drive during cough in the cat (Bolser et al., 1994). In these previous studies, PS inspiratory muscle EMG activity was not recorded. The present study shows that a higher i.a. dose of this drug does result in suppression of inspiratory motor drive to PS spinal inspiratory motoneuron pools during cough. This motoneuron pool can be subject to differential regulation during non-breathing postural behaviors in humans relative to other inspiratory muscles (Butler et al., 2014). The extent to which pharmacological interventions can have differential actions on diaphragm and PS motoneuron activity during coughing is not known.

Baclofen has been historically used to treat spasticity in spinal cord injury and cerebral palsy patients *via* i.t. administration (Albright et al., 1991; Penn et al., 1989). The lack of effect of this drug on cough-related or expiratory load induced increases in abdominal motor drive was somewhat surprising. However, the fact that the abdominal muscle EMG response to noxious pinch of the tail was suppressed by i.t. baclofen indicates that the doses selected for the study were sufficient to decrease non-cough motor responses controlled by spinal circuitry (Kato et al., 1978; Straube et al., 2014). Therefore, the lack of effect of baclofen on abdominal cough-related EMG magnitudes are unlikely to be explained by subthreshold i.t. dosing.

Premotor neurons to spinal abdominal muscle motoneurons are located in the caudal ventral respiratory column in the cat, which corresponds to the nucleus retroambigualis (Iscoe, 1998). We have reported that microinjection of codeine into this area did not suppress abdominal motor drive that was induced by microinjection of the excitatory amino acid agonist, D,L-homocysteic acid, into the same location (Poliacek et al., 2010). In the current study, we found that abdominal motor drive that was induced by expiratory threshold loads was not affected by either i.t. or i.a. baclofen or codeine, although i.a. administration of these drugs did alter cough-related abdominal motor drive. Increases in abdominal motor drive due to respiratory loading are due to decreased pulmonary volume-related feedback and prolongation of the expiratory phase of breathing, leading to a higher peak drive of expiratory premotor neurons in the caudal medulla (Bajic et al., 1992). These findings support our previous conclusion (Poliacek et al., 2010) that suppression of abdominal motor drive during cough by centrally-acting antitussive drugs is likely to be due to actions on neurons other than expiratory premotor neurons in the medulla. We also suggest that activation of expiratory spinal motor pathways by perturbation of slowly adapting receptor activity (*via* respiratory loading) is due to brainstem and spinal pathways that are relatively insensitive to cough suppressants.

## Data Availability Statement

The raw data supporting the conclusions of this article will be made available by the authors, without undue reservation.

## Ethics Statement

The animal study was reviewed and approved by the University of Florida Animal Care and Use Committee.

## Author Contributions

WO analyzed and interpreted data with DB, prepared and edited the manuscript, prepared the graphs and table, and submitted the manuscript for peer review. MR conducted experiments, analyzed data, collected data, and assisted with manuscript preparation. FG, CW, and JH assisted with conducting experiments and data collection. DB is the senior author who conceived the protocol, assisted with interpretation of data, and manuscript preparation. All authors contributed to the article and approved the submitted version.

## Conflict of Interest

The authors declare that the research was conducted in the absence of any commercial or financial relationships that could be construed as a potential conflict of interest.

## References

[B1] Albright A. L. Cervi A. Singletary J. (1991). Intrathecal Baclofen for Spasticity in Cerebral-Palsy. *Jama-J. Am. Med. Assoc.* 265 1418–1422. 10.1001/jama.265.11.1418 1999883

[B2] Aylward M. Maddock J. Davies D. E. Protheroe D. A. Leideman T. (1984). Dextromethorphan and codeine: comparison of plasma kinetics and antitussive effects. *Eur. J. Respir. Dis.* 65 283–291. 6539224

[B3] Baekey D. M. Morris K. F. Nuding S. C. Segers L. S. Lindsey B. G. Shannon R. (2003). Medullary raphe neuron activity is altered during fictive cough in the decerebrate cat. *J. Appl. Physiol.* 94 93–100. 10.1152/japplphysiol.00341.2002 12486018

[B4] Bajic J. Zuperku E. J. Tonkovic-Capin M. Hopp F. A. (1992). Expiratory bulbospinal neurons of dogs. I. Control of discharge patterns by pulmonary stretch receptors. *Am. J Physiol.* 262 R1075–R1086. 1621861 10.1152/ajpregu.1992.262.6.R1075

[B5] Basmajian J. V. Stecko G. A. (1962). New Bipolar Electrode for Electromyography. *J. Appl. Physiol.* 17 849–849. 10.1152/jappl.1962.17.5.849

[B6] Bolser D. C. (2006). Current and future centrally acting antitussives. *Respir. Physiol. Neurobiol.* 152 349–355. 10.1016/j.resp.2006.01.015 16517221 PMC3131070

[B7] Bolser D. C. (1985). Fictive cough in the cat. *J. Appl. Physiol.* 71:1991. 10.1152/jappl.1991.71.6.2325 1778930

[B8] Bolser D. C. Aziz S. M. DeGennaro F. C. Kreutner W. Egan R. W. Siegel M. I. (1993). Antitussive effects of GABAB agonists in the cat and guinea-pig. *Br. J. Pharmacol.* 110 491–495. 10.1111/j.1476-5381.1993.tb13837.x 8220912 PMC2175970

[B9] Bolser D. C. Davenport P. W. (2007). Codeine and cough: an ineffective gold standard. *Curr. Opin. Allergy Clin. Immunol.* 7 32–36. 10.1097/aci.0b013e3280115145 17218808 PMC2921574

[B10] Bolser D. C. Davenport P. W. (2002). Functional organization of the central cough generation mechanism. *Pulm. Pharmacol. Ther.* 15 221–225. 10.1006/pupt.2002.0361 12099768

[B11] Bolser D. C. DeGennaro F. C. (1994). Effect of codeine on the inspiratory and expiratory burst pattern during fictive cough in cats. *Brain Res.* 662 25–30. 10.1016/0006-8993(94)90792-7 7859078

[B12] Bolser D. C. DeGennaro F. C. Chapman R. W. Hey J. A. (1995). “Central and peripheral sites of action of antitussive drugs in the cat”. In *Ventral Brainstem Mechanisms and Control Functions Lung Biology in Health and Disease.* Lenfant C. New York, NY: Marcel Dekker, 95–102.

[B13] Bolser D. C. DeGennaro F. C. O’Reilly S. Chapman R. W. Kreutner W. Egan R. W. (1994). Peripheral and central sites of action of GABA-B agonists to inhibit the cough reflex in the cat and guinea pig. *Br J Pharmacol* 113 1344–1348. 10.1111/j.1476-5381.1994.tb17145.x 7889290 PMC1510532

[B14] Bolser D. C. DeGennaro F. C. O’Reilly S. McLeod R. L. Hey J. A. (1997). Central antitussive activity of the NK1 and NK2 tachykinin receptor antagonists, CP-99,994 and SR 48968, in the guinea-pig and cat. *Br. J. Pharmacol.* 121 165–170. 10.1038/sj.bjp.0701111 9154323 PMC1564671

[B15] Bolser D. C. Hey J. A. Chapman R. W. (1999). Influence of central antitussive drugs on the cough motor pattern. *J. Appl. Physiol.* 86 1017–1024. 10.1152/jappl.1999.86.3.1017 10066718

[B16] Bolser D. C. Poliacek I. Jakus J. Fuller D. D. Davenport P. W. (2006). Neurogenesis of cough, other airway defensive behaviors and breathing: A holarchical system? *Respir. Physiol. Neurobiol.* 152 255–265. 10.1016/j.resp.2006.01.008 16723284 PMC3121153

[B17] Bongianni F. Mutolo D. Fontana G. A. Pantaleo T. (1998). Discharge patterns of Botzinger complex neurons during cough in the cat. *Am. J. Physiol.* 274 R1015–R1024. 9575964 10.1152/ajpregu.1998.274.4.R1015

[B18] Bowery N. G. (1982). Baclofen - 10 Years On. *Trends Pharmacol. Sci.* 3 400–403. 10.1016/0165-6147(82)91205-6

[B19] Butler J. E. Hudson A. L. Gandevia S. C. (2014). The neural control of human inspiratory muscles. *Prog. Brain Res.* 295–308. 10.1016/b978-0-444-63274-6.00015-1 24746054

[B20] Canning B. J. Mori N. Lehmann A. (2012). Antitussive effects of the peripherally restricted GABAB receptor agonist lesogaberan in guinea pigs: comparison to baclofen and other GABAB receptor-selective agonists. *Cough* 8:7. 10.1186/1745-9974-8-7 23025757 PMC3520872

[B21] Castillo D. Pitts T. (2013). Influence of baclofen on laryngeal and spinal motor drive during cough in the anesthetized cat. *Laryngoscope* 123 3088–3092. 10.1002/lary.24143 23670824 PMC4936775

[B22] Chou D. T. Wang S. C. (1975). Studies on the localization of central cough mechanism; site of action of antitussive drugs. *J. Pharmacol. Exp. Ther.* 194 499–505. 1159627

[B23] Dicpinigaitis P. V. (2009). Currently available antitussives. *Pulm. Pharmacol. Ther.* 22 148–151. 10.1016/j.pupt.2008.08.002 18771744

[B24] Dicpinigaitis P. V. Dobkin J. B. Rauf K. (1997). Comparison of the antitussive effects of codeine and the GABA-agonist baclofen. *Clin. Drug Invest.* 14 326–329. 10.2165/00044011-199714040-00012 24715533

[B25] Dicpinigaitis P. V. Dobkin J. B. Rauf K. Aldrich T. K. (1998). Inhibition of capsaicin-induced cough by the gamma-aminobutyric acid agonist baclofen. *J. Clin. Pharmacol.* 38 364–367. 10.1002/j.1552-4604.1998.tb04436.x 9590464

[B26] Dicpinigaitis P. V. Morice A. H. Birring S. S. McGarvey L. Smith J. A. Canning B. J. (2014). Antitussive drugs–past, present, and future. *Pharmacol. Rev.* 66 468–512. 10.1124/pr.111.005116 24671376 PMC11060423

[B27] Gruhzit C. C. (1957). Chemoreflex activity of dextromethorphan (romilar), dextrorphan, codeine and morphine in the cat and the dog. *J. Pharmacol. Exp. Ther.* 120 399–407. 13476364

[B28] Iscoe S. (1998). Control of abdominal muscles. *Prog. Neurobiol.* 56 433–506. 10.1016/s0301-0082(98)00046-x 9775401

[B29] Kato M. Waldmann U. Murakami S. (1978). Effects of baclofen on spinal neurones of cats. *Neuropharmacology* 17 827–833. 10.1016/0028-3908(78)90071-0 745680

[B30] Keller J. A. McGovern A. E. Mazzone S. B. (2017). Translating Cough Mechanisms Into Better Cough Suppressants. *Chest* 152 833–841. 10.1016/j.chest.2017.05.016 28552543

[B31] Korpás J. Tomori Z. N. (1979). Cough and other respiratory reflexes. *Prog. Respir. Res. Basel* 12:356.

[B32] May A. J. Widdicombe J. G. (1954). Depression of the cough reflex by pentobarbitone and some opium derivatives. *Br. J. Pharmacol. Chemother.* 9 335–340. 10.1111/j.1476-5381.1954.tb01689.x 13199253 PMC1509403

[B33] Mazzone S. B. McGovern A. E. Cole L. J. Farrell M. J. (2011). Central nervous system control of cough: pharmacological implications. *Curr. Opin. Pharmacol.* 11 265–271. 10.1016/j.coph.2011.05.005 21664870

[B34] Mazzone S. B. McGovern A. E. Farrell M. J. (2015). Endogenous central suppressive mechanisms regulating cough as potential targets for novel antitussive therapies. *Curr. Opin. Pharmacol.* 22 1–8. 10.1016/j.coph.2015.02.002 25704497

[B35] Mazzone S. B. McGovern A. E. Koo K. Farrell M. J. (2009). Mapping supramedullary pathways involved in cough using functional brain imaging: comparison with pain. *Pulm. Pharmacol. Ther.* 22 90–96. 10.1016/j.pupt.2008.08.003 18804546

[B36] Mazzone S. B. McGovern A. E. Yang S. K. Woo A. Phipps S. Ando A. (2013). Sensorimotor circuitry involved in the higher brain control of coughing. *Cough* 9:7. 10.1186/1745-9974-9-7 23497672 PMC3602068

[B37] Mazzone S. B. McLennan L. McGovern A. E. Egan G. F. Farrell M. J. (2007). Representation of capsaicin-evoked urge-to-cough in the human brain using functional magnetic resonance imaging. *Am. J. Respir. Crit. Care Med.* 176 327–332. 10.1164/rccm.200612-1856oc 17575093

[B38] McGovern A. E. Davis-Poynter N. Yang S. K. Simmons D. G. Farrell M. J. Mazzone S. B. (2015a). Evidence for multiple sensory circuits in the brain arising from the respiratory system: an anterograde viral tract tracing study in rodents. *Brain Struct. Funct.* 220 3683–3699. 10.1007/s00429-014-0883-9 25158901

[B39] McGovern A. E. Driessen A. K. Simmons D. G. Powell J. Davis-Poynter N. Farrell M. J. (2015b). Distinct brainstem and forebrain circuits receiving tracheal sensory neuron inputs revealed using a novel conditional anterograde transsynaptic viral tracing system. *J. Neurosci. Official J. Soc. Neurosci.* 35 7041–7055. 10.1523/jneurosci.5128-14.2015 25948256 PMC6605260

[B40] Mutolo D. (2017). Brainstem mechanisms underlying the cough reflex and its regulation. *Respir. Physiol. Neurobiol.* 243 60–76. 10.1016/j.resp.2017.05.008 28549898

[B41] Mutolo D. Bongianni F. Cinelli E. Fontana G. A. Pantaleo T. (2008). Modulation of the cough reflex by antitussive agents within the caudal aspect of the nucleus tractus solitarii in the rabbit. *Am. J. Physiol. Regul. Integr. Comp. Physiol.* 295 R243–R251. 18480245 10.1152/ajpregu.00184.2008

[B42] Mutolo D. Bongianni F. Cinelli E. Pantaleo T. (1985). Depression of cough reflex by microinjections of antitussive agents into caudal ventral respiratory group of the rabbit. *J. Appl. Physiol.* 109:2010. 10.1152/japplphysiol.00406.2010 20651222

[B43] Ohi Y. Yamazaki H. Takeda R. Haji A. (2005). Functional and morphological organization of the nucleus tractus solitarius in the fictive cough reflex of guinea pigs. *Neurosci. Res.* 53 201–209. 10.1016/j.neures.2005.06.016 16040147

[B44] Penn R. D. Savoy S. M. Corcos D. Latash M. Gottlieb G. Parke B. (1989). Intrathecal Baclofen for Severe Spinal Spasticity. *N. Engl. J. Med.* 320 1517–1521. 2657424 10.1056/NEJM198906083202303

[B45] Plumb D. C. (2011). *Plumb’s Veterinary Drug Handbook.* Stockholm: Wisconsin Pharma Vet Inc.

[B46] Poliacek I. Rose M. J. Pitts T. E. Mortensen A. Corrie L. W. Davenport P. W. (1985). Central administration of nicotine suppresses tracheobronchial cough in anesthetized cats. *J. Appl. Physiol.* 118:2015. 10.1152/japplphysiol.00075.2014 PMC4312848 25477349

[B47] Poliacek I. Simera M. Veternik M. Kotmanova Z. Bolser D. C. Machac P. (2017). Role of the dorsomedial medulla in suppression of cough by codeine in cats. *Respir Physiol Neurobiol* 246 59–66. 10.1016/j.resp.2017.07.011 28778649 PMC5646267

[B48] Poliacek I. Simera M. Veternik M. Machac P. Barani H. Visnovcova N. (2012). Contribution of medullary raphé to control of coughing–codeine trials in cat. *Respir. Physiol. Neurobiol.* 184 106–112. 10.1016/j.resp.2012.08.010 22929585

[B49] Poliacek I. Wang C. Corrie L. W. Rose M. J. Bolser D. C. (2010). Microinjection of codeine into the region of the caudal ventral respiratory column suppresses cough in anesthetized cats. *J. Appl. Physiol.* 108 858–865. 10.1152/japplphysiol.00783.2009 20093669 PMC2853207

[B50] Ross B. B. Gramiak R. Rahn H. (1955). Physical dynamics of the cough mechanism. *J. Appl. Physiol.* 8 264–268. 10.1152/jappl.1955.8.3.264 13271252

[B51] Satia I. Badri H. Al-Sheklly B. Smith J. A. Woodcock A. A. (2016). Towards understanding and managing chronic cough. *Clin. Med.* 16 s92–s97. 10.7861/clinmedicine.16-6s-s92 PMC6329565 27956447

[B52] Schappert S. M. (1999). Ambulatory care visits to physician offices, hospital outpatient departments, and emergency departments: United States, 1997. *Vital Health Statist. Series* 13 1–39. 10633576

[B53] Shannon R. Baekey D. Morris K. Lindsey B. (1996). Brainstem respiratory networks and cough. *Pulm. Pharmacol.* 9 343–347. 10.1006/pulp.1996.0045 9232673

[B54] Shannon R. Baekey D. Morris K. Nuding S. Segers L. Lindsey B. (2004a). Production of reflex cough by brainstem respiratory networks. *Pulm. Pharmacol. Ther.* 17 369–376. 10.1016/j.pupt.2004.09.022 15564078

[B55] Shannon R. Baekey D. M. Morris K. F. Li Z. Lindsey B. G. (2000). Functional connectivity among ventrolateral medullary respiratory neurones and responses during fictive cough in the cat. *J.Physiol.* 525(Pt 1), 207–224. 10.1111/j.1469-7793.2000.00207.x 10811738 PMC2269920

[B56] Shannon R. Baekey D. M. Morris K. F. Lindsey B. G. (1998). Ventrolateral medullary respiratory network and a model of cough motor pattern generation. *J. Appl. Physiol.* 84 2020–2035. 10.1152/jappl.1998.84.6.2020 9609797

[B57] Shannon R. Baekey D. M. Morris K. F. Nuding S. C. Segers L. S. Lindsey B. G. (2004b). Pontine respiratory group neuron discharge is altered during fictive cough in the decerebrate cat. *Respir. Physiol. Neurobiol.* 142 43–54. 10.1016/j.resp.2004.05.002 15351303

[B58] Simera M. Poliacek I. Jakus J. (2010). Central Antitussive Effect of Codeine in the Anesthetized Rabbit. *Eur. J. Med. Res.* 15 184–188. 21147648 10.1186/2047-783X-15-S2-184 PMC4360299

[B59] Smith J. Owen E. Earis J. Woodcock A. (2006). Effect of codeine on objective measurement of cough in chronic obstructive pulmonary disease. *J. Allergy Clin. Immunol.* 117 831–835. 10.1016/j.jaci.2005.09.055 16630941

[B60] Smith J. A. Hilton E. C. Y. Saulsberry L. Canning B. J. (2012). Antitussive effects of memantine in guinea pigs. *Chest* 141 996–1002. 10.1378/chest.11-0554 22016492 PMC3318948

[B61] Straube C. Derry S. Jackson K. C. Wiffen P. J. Bell R. F. Strassels S. (2014). Codeine, alone and with paracetamol (acetaminophen), for cancer pain. *Cochrane Database Syst. Rev.* 2014:CD006601. 10.1002/14651858.CD006601.pub4 PMC6513650 25234029

[B62] Wang Z. Logothetis N. K. Liang H. (2009). Spatiotemporal neural integration for bistable perception in a response-time structure-from-motion task. *IEEE Trans Biomed. Eng.* 56 2937–2948. 10.1109/tbme.2009.2027332 19635689

[B63] Widdicombe J. G. (1980). Mechanism of Cough and Its Regulation. *Eur. J. Respir. Dis.* 61 11–20. 7011828

[B64] Xu F. Frazier D. T. Zhang Z. Baekey D. M. Shannon R. (1985). Cerebellar modulation of cough motor pattern in cats. *J. Appl. Physiol.* 83:1997. 10.1152/jappl.1997.83.2.391 9262432

